# Location and Pathogenic Potential of *Blastocystis* in the Porcine Intestine

**DOI:** 10.1371/journal.pone.0103962

**Published:** 2014-08-05

**Authors:** Wenqi Wang, Helle Bielefeldt-Ohmann, Rebecca J. Traub, Leigh Cuttell, Helen Owen

**Affiliations:** 1 School of Veterinary Science, The University of Queensland, Gatton, Queensland, Australia; 2 Australian Infectious Diseases Research Centre, The University of Queensland, St. Lucia, Queensland, Australia; 3 Faculty of Veterinary Science, The University of Melbourne, Parkville, Victoria, Australia; UNIFESP Federal University of São Paulo, Brazil

## Abstract

*Blastocystis* is an ubiquitous, enteric protozoan of humans and many other species. Human infection has been associated with gastrointestinal disease such as irritable bowel syndrome, however, this remains unproven. A relevant animal model is needed to investigate the pathogenesis/pathogenicity of *Blastocystis*. We concluded previously that pigs are likely natural hosts of *Blastocystis* with a potentially zoonotic, host-adapted subtype (ST), ST5, and may make suitable animal models. In this study, we aimed to characterise the host-agent interaction of *Blastocystis* and the pig, including localising *Blastocystis* in porcine intestine using microscopy, PCR and histopathological examination of tissues. Intestines from pigs in three different management systems, i.e., a commercial piggery, a small family farm and a research herd (where the animals were immunosuppressed) were examined. This design was used to determine if environment or immune status influences intestinal colonisation of *Blastocystis* as immunocompromised individuals may potentially be more susceptible to blastocystosis and development of associated clinical signs. Intestines from all 28 pigs were positive for *Blastocystis* with all pigs harbouring ST5. In addition, the farm pigs had mixed infections with STs 1 and/or 3. *Blastocystis* organisms/DNA were predominantly found in the large intestine but were also detected in the small intestine of the immunosuppressed and some of the farm pigs, suggesting that immunosuppression and/or husbandry factors may influence *Blastocystis* colonisation of the small intestine. No obvious pathology was observed in the histological sections. *Blastocystis* was present as vacuolar/granular forms and these were found within luminal material or in close proximity to epithelial cells, with no evidence of attachment or invasion. These results concur with most human studies, in which *Blastocystis* is predominantly found in the large intestine in the absence of significant organic pathology. Our findings also support the use of pigs as animal models and may have implications for blastocystosis diagnosis/treatment.

## Introduction

*Blastocystis* is an ubiquitous, intestinal protozoan that was first described by A. Alexieff in 1911, and subsequently found to infect a variety of hosts including humans and many animal species [1]–[4]. A century later, it still remains an enigmatic organism with controversy over its life cycle and most importantly, pathogenicity. In humans, the association between *Blastocystis* infection and gastrointestinal disease remains controversial [4]. A relationship with irritable bowel syndrome (IBS) has been suggested in several studies [5]–[8], often based on a higher *Blastocystis* prevalence in IBS patients compared to control groups. The proposed mechanism of *Blastocystis-*associated IBS is low grade inflammation of the mucosa, which is well recognised in IBS patients, associated with persistent *Blastocystis* infection and antigenic stimulation [7]. However, more extensive, controlled studies need to be conducted to clearly define the role of *Blastocystis,* if any, in gastrointestinal disease and IBS [7].

In recent years, intestinal protozoa have been shown to cause increased morbidity and mortality in immunocompromised hosts (e.g. human immunodeficiency virus (HIV) patients, transplant patients, immunocompromised children) [9]–[11]. A link between immunosuppression and blastocystosis has been proposed, with immunocompromised individuals, such as patients infected with HIV, cancer patients, renal transplant patients and children, potentially being more susceptible to infection and associated clinical signs [4], [10], [12]–[14]. We speculate that *Blastocystis* could be an opportunistic, gastrointestinal pathogen and that immunosuppression may be an important predisposing factor for disease development.

One of the major obstacles to understanding the pathogenicity of the organism and the pathogenesis of blastocystosis is the lack of an established animal model to fulfil Koch’s postulates [4], [7], [15]. Experimental infections have been conducted in guinea pigs, mice, chickens and rats with conflicting results on pathogenic potential of *Blastocystis*. Mild pathology and self-limiting disease has been observed in mice, while moderate to severe pathology has been observed in some chickens and rats with various *Blastocystis* isolates [16]–[19]. Reported histopathological findings in these animal infectivity studies include inflammation, mucosal sloughing and mild lamina propria oedema. *Blastocystis* organisms were usually found in luminal material or at the epithelial edge in the caecum and colon of infected mice [17]. To date, conclusive evidence of *Blastocystis-*associated histopathology in human intestine is lacking, therefore, most of these animal models are unlikely to be representative of host-adapted subtype (ST) infection in a human host.

In a previous study we reported a high prevalence of *Blastocystis* carriage in pigs (up to 76.7%) with all pigs harbouring ST5 and a small proportion of pigs harbouring STs 1 and/or 3 [20]. Based on these results, we concluded that pigs are likely to be a natural host of *Blastocystis* with ST5 being the host adapted ST [20]. In addition, they are able to harbour STs 1 and 3, the two most commonly reported STs in humans [1], and therefore may be suitable candidates for animal model development.

The first aim of the current study was to localise *Blastocystis* in the intestinal tract of pigs. While *Blastocystis* has been demonstrated in the caecum and colon in animal infectivity studies [17], [18], there have been no definitive reports of site predilection in humans. Secondly, we aimed to assess porcine intestine for any colonisation associated histopathology and compare with the human paradigm where significant organic pathology is usually not identified. Lastly, we aimed to determine if immune status and/or environmental factors influence the pathogenicity of *Blastocystis* in pigs.

## Materials and Methods

### Sampling

Intestinal samples were collected during post-mortem examination of 28 pigs from three locations in Southeast Queensland, Australia: 1) 12 healthy pigs from a small family farm, 2) 11 pigs from a commercial intensive piggery (6 weaners and 5 growers/sows) and, 3) five grower pigs from a research facility. The research pigs were originally obtained as piglets from the commercial piggery before being transferred to the research facility where they were early weaned at three weeks and treated daily with 0.25 mg/kg of oral Dexamethasone until 16 weeks of age to induce immunosuppression. These research pigs were fed on a commercial diet with no added antibiotics. Commercial pigs were given an amoxycillin injection at weaning (4 weeks of age) and transferred to a commercial feed which differed from that given to the research pigs. Feed for the commercial pigs was medicated with olaquindox till 9 weeks of age. From 9 weeks onwards, antibiotics were given only when animals were ill. This indicates that the 6 commercial weaners were on antibiotics whilst the 5 commercial growers/sows were not.

Post-mortem samples were collected after the pigs had either died of causes unrelated to the gastrointestinal tract (e.g. neurological signs, sudden death) or were healthy pigs that were slaughtered at the Brisbane Valley Meats abattoir (Queensland, Australia) or euthanised after unrelated research or teaching use. Samples were collected at a maximum of 12 hours following death with the carcass being refrigerated for the interval between death and sampling to minimise autolysis.

A variety of samples were collected from the three sections of small intestine, namely duodenum, jejunum and ileum and three sections of large intestine, namely caecum, colon and rectum (Table 1). Samples collected from each intestinal segment included luminal contents, mucosal scrapings and cross sectional tissue biopsies. For mucosal scraping collection, segments of mucosa were first rinsed gently in phosphate buffered saline (PBS) twice and scrapings were taken by gently scraping the washed mucosal surface with a scalpel blade till the top layer dislodged. Luminal contents and mucosal scrapings were stored in individual 50 ml sterile plastic containers and 2 ml microcentrifuge tubes, respectively, at room temperature and processed within 6 hours of collection. Biopsy samples were stored in 10% buffered formalin at room temperature for at least 48 hours before processing.

**Table 1:** Samples collected and tests carried out from each segment of porcine intestine.

	Sample collected		
Technique	Luminal contents	Mucosa scraping	Tissue biopsy
Light microscopy (faecal wet mount)	X	X	
Xenic in-vitro culture*	X		
PCR	X (+ culture material)	X	
Histopathology			X

### Faecal wet mount and xenic in-vitro culture (XIVC)

Fresh luminal material was added to distilled water to make a faecal wet mount and examined for *Blastocystis* organisms using light microscopy. For XIVC, approximately 1–2 grams of luminal material was incubated with 10 ml of Jones’ culture medium enriched with 10% heat-inactivated horse serum (Gibco, 26050088, Life Technologies, USA) at 37°C under aerobic conditions for 24–48 hours and examined using light microscopy [21], [22].

### Molecular analysis

#### i. DNA extraction

DNA was extracted from fresh luminal material, culture material and mucosal scrapings using QIAamp DNA Stool Mini Kit (Qiagen Germany), as previously described [23].

#### ii. PCR amplification with nested PCR primers

Samples were initially tested with a published pan*-Blastocystis* nested PCR primer set and conditions, amplifying a 1100 bp region of the *Blastocystis* 18S ribosomal small subunit RNA gene (18S SSU rRNA gene) [24], [25]. Each 25 µl PCR reaction was run with approximately 50 ng of DNA.

#### iii. Phylogenetic analysis to subtype *Blastocystis* isolates

PCR products of samples positive with the nested PCR were purified with the PureLink Genomic DNA Mini Kit (Life Technologies Corporation, New York, USA) according to the manufacturer’s protocol. Using an Applied Biosystems 3130/3130xl Genetic Analyzer, unidirectional DNA sequencing was performed on purified products using the reverse primer of the secondary PCR. The sequences were then analysed using Finch TV v 1.4.0 (Geospiza Inc., Seattle, WA, USA) and compared with published sequences from GenBank (National Center for Biotechnology Information) using BLAST 2.2.9 (http://blast.ncbi.nlm.nih.gov/Blast.cgi). Utilising Bioedit c 7.1.3.0 software (Ibis Biosciences, Carlsbad, CA, USA), these sequences were aligned with published sequences of the 18S SSU rRNA gene of the various *Blastocystis* STs, which were sourced from GenBank. The aligned sequences were then analysed using Mega 4.1 software (The Biodesign Institute, Tempe, AZ, USA) to construct a neighbour joining tree, with *Proteromonas lacerate* (U37108) as an out-group. This was performed to subtype the *Blastocystis* isolates.

#### iv. PCR amplification with ST-specific primers

Published subtype-specific (ST-specific) primers for STs 1, 3 and 5 were used to test samples with suspected mixed ST infections as these STs were previously observed in this population of pigs and in-contact humans [20]. These suspected mixed ST infections were identified using the nested PCR described above (i.e., double peaks observed during sequence analysis). Each of these ST-specific primers was designed to amplify partial segments of the 18S SSU rRNA gene of *Blastocystis*.

### Histopathological analysis of biopsies

**Figure 1:**
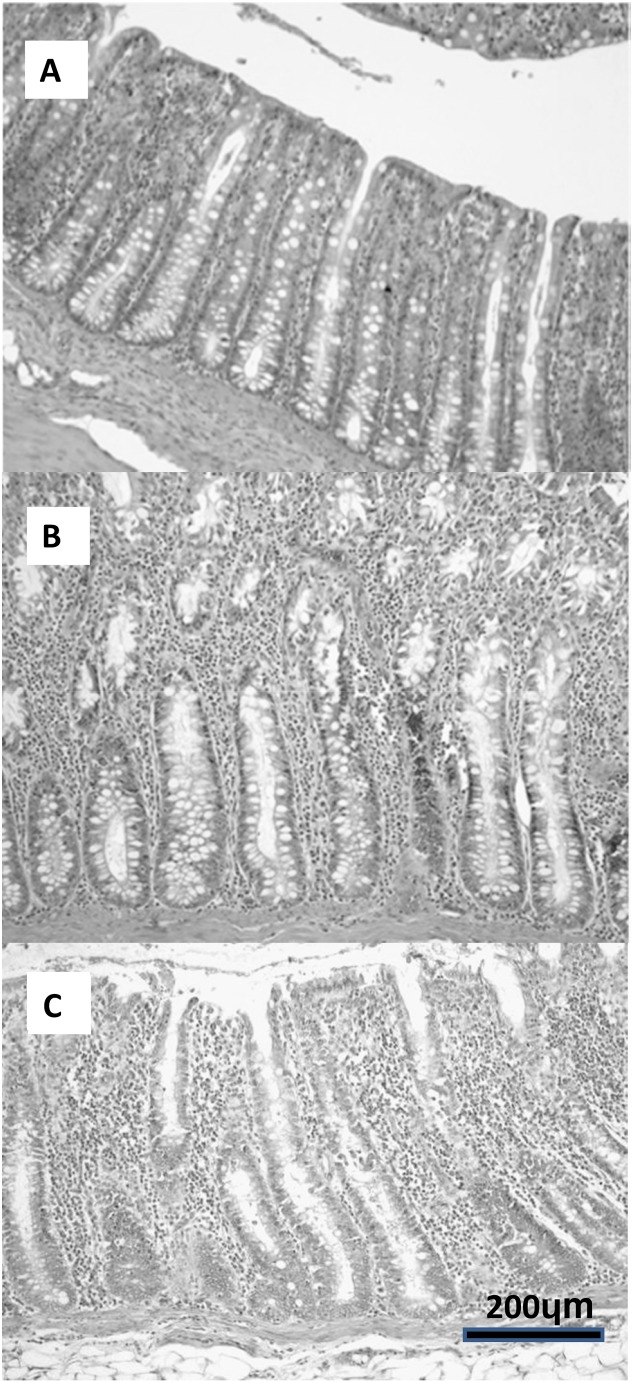
Grading scheme for quantifying leucocytes in lamina propria of porcine intestine. Haematoxylin and eosin (10X). (A) low: 1–3 leucocytes between crypts, (B) medium: 3 to 5 leucocytes between crypts, (C) high: >5 leucocytes between crypts.

Cross-sectional tissue biopsies were taken from each intestinal segment and fixed in 10% buffered formalin for at least 48 hours. Only segments positive by PCR were processed for histological analysis. Tissue sections were cut at 5 µm, stained with haematoxylin and eosin and examined using light microscopy. Slides were examined for presence and location of *Blastocystis* in the mucosa, evidence of mucosal damage, inflammation or increased numbers of infiltrating leucocytes in the mucosa or any other associated pathology. The leucocyte population in the lamina propria was characterised by determining average number and type of leucocytes between the intestinal crypts (e.g., lymphocytes, plasma cells, neutrophils, eosinophils etc.) (Figure 1, grading scheme). The grading scheme was based on a grading scheme developed for dog and cat intestinal biopsies [26]. Each pig was given an average histological grade for small and large intestine, respectively, by summing up the grade for the three segments (if applicable) in large or small intestine. As controls, several PCR negative small and large intestine samples were used for comparison.

### Statistical analysis

Statistical analysis was carried out using either the program WINPEPI [27] or an online program http://quantpsy.org [28]. These two programs were used to perform Pearson’s chi-squared tests to: 1) determine if any ST was associated with any particular intestinal segment [28] and 2) compare the probability of pigs from each of the three different groups having *Blastocystis* in the small intestine [27].

### Ethics statement

This project was approved by the University of Queensland Animal Ethics Committee and Medical Research Ethics Committee with approval no. ANRFA/SVS/347/12.

## Results and Discussion

### Molecular detection and localisation of *Blastocystis*

All (28/28) pigs, regardless of origin, were positive for *Blastocystis* by microscopy and PCR and all animals harboured ST5 (Table 2). Pigs from the commercial piggeries and research facility harboured ST5 only (Table 3). In contrast, 7/12 (58%) of the pigs from the small scale farm had mixed infections of ST5 and ST1 and/or 3, with the remaining 5 harbouring ST5 only (Table 3). The STs found in this cohort of pigs corresponded to those found in our previous study [20]. All pigs were consistently found to harbour *Blastocystis* in the large intestine by XIVC and PCR of faecal and cultured material, with *Blastocystis* being found in all the colons and approximately 90% of the caeca and rectums examined (Table 3). Analysis using Pearson’s chi-squared tests demonstrated that no ST was significantly associated with a particular intestinal location (*p*-value for ST1, ST3 and ST5 respectively were >0.05) [28]. Our results concur with several other studies, including a study on naturally infected pigs where *Blastocystis* was mainly found in the caecum and colon using immunofluorescence assay (IFA) in combination with PCR [29]. Previous experimental animal infectivity studies also found *Blastocystis* mainly in the caecum and colon of mice, guinea pigs and rats; however some of these animals had undergone intracaecal inoculation with *Blastocystis* [17]–[19]. This finding supports the potential link between IBS and *Blastocystis* as IBS is considered a condition that affects the large bowel/colon.

**Table 2:** Frequency of detection of *Blastocystis* in porcine intestinal segments using PCR (nested and ST-specific primers) in small versus large intestine.

	Intestinal Section	
Farm	Small and large intestine	Large intestine only
**Commercial (11)**	1	10
**Research (5)**	5	0
**Small family farm (12)**	4	8
**Total no. of pigs (28)**	10	18

**Table 3:** Frequency of detection and ST distribution of *Blastocystis* in porcine intestinal segments using PCR (nested and ST-specific primers) in intestinal segments.

	Intestinal segment						
Farm	ST	Duo	Jej	Ile	Cae	Col	Rec
**Total no.** **of pigs (28)**	**Any**	**4**	**5**	**7**	**25**	**28**	**24** *
**Commercial (11)**	**ST 5**	**2**	**0**	**0**	**10**	**11**	**8**
**Research (5)**	**ST 5**	**1**	**3**	**5**	**5**	**5**	**5**
**Small family farm (12)**	**Any ST** **	**1**	**2**	**2**	**10**	**12**	**11**
	**ST 1**	0	2	2	4	5	5
	**ST 3**	1	1	1	4	5	5
	**ST 5**	1	1	0	7	11	11

Duo: duodenum, Jej: jejunum, Ile: ileum, Cae: caecum, Col: colon, Rec: rectum.

*We were unable to obtain rectal samples from 2 pigs, 1 each from the commercial piggery and small family farm respectively.

**In the commercial piggery and research facility only ST5 was detected whereas STs 1, 3 and 5 were detected in the small outdoor farm. Seven out of 12 of the pigs from the small outdoor farm had mixed infections of ST 5 and ST1 and/or 3.

In contrast, *Blastocystis* DNA was detected within the small intestine in only 36% (10/28) of the pigs (Table 2, 3). Of these 10 pigs, 1 (9.1%) was from a commercial piggery, 4 (33.3%) from the small scale piggery and the remaining 5 (100%) were immunosuppressed research pigs (Table 3). Statistical comparison of different environmental settings showed that immunosuppressed research pigs were much more likely to have *Blastocystis* DNA detected in the small intestine as compared to the commercial and farm pigs respectively (Table 4; *p-*value = <0.05, odds ratio = 0.00, reciprocal ∞). In the research pigs, *Blastocystis* DNA was not detected in the duodenum, however it was found in the jejunum of 3/5 animals and in the ileum of 5/5 pigs (Table 3).

**Table 4:** Chi-Square analysis of proportion of pigs in each farm setting with *Blastocystis* DNA detected in the small intestine.

Farm	Pearson’s Chi Squarevalue (χ^2^)	*p*-value	Odds ratio (95% C.I)
**Commercial vs** 1 **Research**	?^2^ = 12.121;	0.000	0, reciprocal ∞ (95% C.I. = 0.00 to 0.33)
**Small family farm vs Research**	?^2^ = 6.296;	0.012	0, reciprocal ∞ (95% C.I. = 0.00 to 0.92)
**Commercial vs Small family farm**	?^2^ = 1.982;	0.159	0.2, reciprocal 5.00 (95% C.I. = 0.00 to 2.75)

1 vs-versus.

Reported effects of immunosuppression on the gastrointestinal (GIT) system include loss of gastric acidity, impaired/deficient T cell mediated immune response, reduced mucosal integrity and impaired mucosal regeneration [10], [30]. Impaired T cell immune response has been shown to facilitate infection of the immunocompromised host with intestinal protozoa, however ultimately the pathogenesis within the host is not clearly understood [10]. It is possible that the dexamethasone-induced immunosuppression in these pigs had led to functional pathology such as a dysregulated T cell-mediated immune response [31], [32]. These effects may then allow *Blastocystis* to colonise not only the usual locations in the large intestine but to also ascend into the distal sections of the small intestine, where it could have a different interaction with the host and potentially cause disease (opportunistic infection). It should be noted that in this study we were unable to evaluate the immune system of the dexamethasone-treated pigs. However, these pigs were used primarily for a project investigating the immunopathology of scabies. The pilot study of this scabies project showed that using the above mentioned dexamethasone regime, dexamethasone-treated mange infected pigs were more likely to be chronically infected as compared to untreated pigs that had no/few mites and symptoms that resolved within 6 weeks [33]. Given that all the commercial pigs, regardless of age and antibiotic regime, had *Blastocystis* predominantly in the large but not small intestine, it is unlikely that the antibiotics had any effect on location of *Blastocystis* colonisation in the porcine intestine.

Amongst the 10 pigs in which *Blastocystis* DNA was detected in the small intestine, 4/10, 5/10 and 7/10 had *Blastocystis* DNA in the duodenum, jejunum and ileum contents respectively. This is in contrast to the study by Fayer et al. [29], where *Blastocystis* was not detected in the ileum, jejunum and duodenum contents using PCR and IFA, and was only detected in small numbers in the jejunum tissue using IFA. The discrepancy between the results of the current study and those of Fayer et al. [29] could be due to a number of factors that could influence sensitivity of detection. These include different samples tested such as cultured material and mucosal scrapings, different diagnostic techniques and PCR primers used (nested PCR and STS primers) which could better sensitivity of detection. This would be especially likely if only few organisms/small amounts of DNA were present in the small intestine, as also suggested by Fayer et al. [29]. Alternatively, the different environmental and housing conditions of the pigs (e.g., immunosuppressed pigs, diet) may have an influence on small intestine survival/colonisation of *Blastocystis*.

In this study, PCR of cultured material was the most sensitive method of *Blastocystis* detection. Setting this method as a gold standard, the sensitivities of XIVC, PCR of luminal material DNA, a wet faecal smear and PCR of mucosal scrapings were 81.3%, 58.5%, 45% and 32.5% respectively. PCR is widely recognised as the gold standard for *Blastocystis* diagnosis and XIVC has almost comparable sensitivity [22], [34]–[36]. We found combining the two techniques in series (amplification of small numbers of *Blastocystis* from luminal material using XIVC followed by PCR) the most sensitive method for detection. Ideally, a larger sample size would allow for more accurate establishment of sensitivity.

In the small intestine of the commercial and small scale piggery pigs, *Blastocystis* DNA was detected mostly in cultured material, whereas in the research pigs it was detected in faeces in 3/5 pigs and in cultured material in 5/5 of the pigs. Taking into account that PCR of cultured material is the most sensitive detection method and the PCR results (Table 2, 3), we speculate that there is generally a lower number of *Blastocystis* organisms/DNA in the small intestine as compared to the large intestine. The low sensitivity of PCR on mucosal scrapings could be due to *Blastocystis* having no or weak attachment to the epithelium and/or due to prior rigorous rinsing of the surface with PBS.

### Histopathology

**Figure 2:**
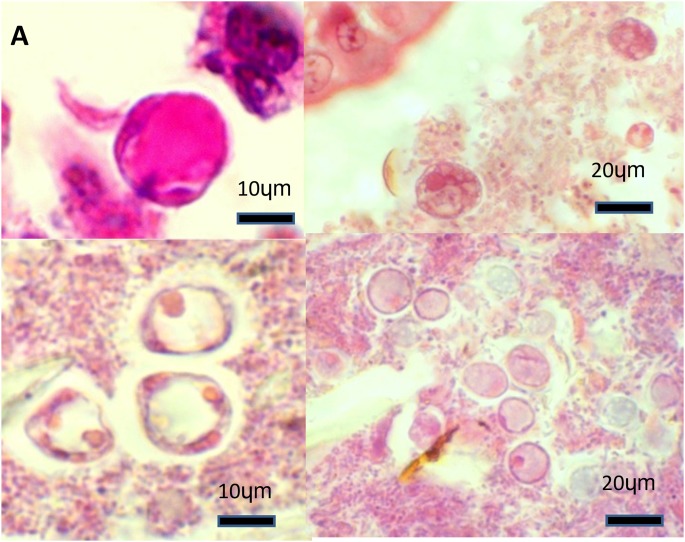
Histological images of *Blastocystis* organisms in the porcine intestinal biopsies. Haematoxylin and eosin. (A) Caecum from a commercial pig: a *Blastocystis* vacuolar form amongst intestinal lumen material (100X). (B) Colon from a research pig: several *Blastocystis* granular forms found near the tip of the intestinal epithelium (40X). (C) Colon from a commercial pig: three *Blastocystis* vacuolar forms of amongst luminal material (100X) (D) Colon from a commercial pig: several vacuolar *Blastocystis* organisms scattered amongst intestinal lumen material (40X).

In the intestinal histological sections examined, granular or vacuolar forms of *Blastocystis* organisms were often observed either within the luminal material or at the tip of the epithelium, but there was no evidence of attachment or invasion of the epithelium (Figure 2). The organisms were most commonly identified in the caecum and colon histological sections, which corresponded to the PCR results. There was no associated pathology such as frank inflammation, epithelial damage, mucosal sloughing or lamina propria oedema as has been described previously in experimental animal infectivity models [17], [18], [37]. This lack of pathology was also observed in the preceding study on naturally infected pigs [29]. Comparison of histological grading of PCR positive small and large intestinal histological sections with the negatives did not demonstrate any large differences in grade (Table 5). Both *Blastocystis* positive and negative groups of pigs had histological grades that ranged from low to low/medium to medium, as such we cannot confidently identify any increase in inflammatory cells associated with *Blastocystis* carriage (Table 5). Amoeboid forms that have been suggested to be pathogenic [38] were not observed in the asymptomatic pigs in this study.

**Table 5:** Average intestinal histological grade for *Blastocystis* positive porcine large intestinal sections in each piggery setting.

	Histological grade				
Farm	Low	Low/medium	Medium	Medium/high	High
Commercial (11)	7	1	1	1	1
Small family farm (12)	-	4	3	3	2
Research (5)	-	5	-	-	-
Total (28)	7	10	4	3	3

The average histological grade for the small intestinal sections in which *Blastocystis* was detected was either low/medium. The average histological grade for the PCR negative control sections for small and large intestine were medium and low/medium respectively.

In this study, the lack of organic pathology in *Blastocystis-*infected porcine intestine concurs with a study in naturally infected pigs [29] and the majority of the earlier human studies, with the exception of 2 case studies. Both patients had ulcerative colitis with associated superficial colonisation by *Blastocystis* organisms [39], [40]. This invasive behaviour is likely the result of the underlying ulcers allowing for opportunistic, superficial colonisation of *Blastocystis*. Although *Blastocystis* has been linked to certain intestinal conditions such as ulcerative colitis and haemorrhagic proctosigmoiditis, no histological evidence of *Blastocystis* causing primary intestinal damage has been demonstrated [41]–[43].

## Conclusions

We propose pigs as suitable animal models for *Blastocystis* as they are natural hosts of *Blastocystis,* with a host adapted ST (ST5), and are able to harbour ST1 and ST3 which are frequently found in humans. Pigs harbour *Blastocystis* predominantly in the large intestine, as detected by molecular and histological methods, which is in agreement with previous animal infectivity studies. Occasionally, *Blastocystis* was detected in the small intestine, notably in the immunosuppressed research pigs, which suggests that immunosuppression may alter host-agent relations and predispose to small intestinal colonisation. Lastly, histological analysis of PCR-positive porcine intestine revealed no evidence of pathology caused by *Blastocystis* which is consistent with the majority of human studies. Alternatively, we could speculate that *Blastocystis* may cause clinical disease via other mechanisms such as host immune modulation or disruption of the epithelial barrier, perhaps in synergism with unknown pathogens or opportunists. In the future, all these aspects can be readily studied in the pig model.
